# GLOSSARY

**Published:** 2004

Acetaldehyde: A toxic product that results from the breakdown of alcohol by the *enzyme alcohol dehydrogenase.*
Adenosine triphosphate (ATP): A molecule that provides the energy needed for many key metabolic reactions. ATP is generated largely in the *mitochondria.*
Alcohol dehydrogenase: An *enzyme* that breaks down alcohol by oxidation, converting it to *acetaldehyde*.
Alcohol poisoning: The result of the acute toxic effects of alcohol consumption, which can range from gastritis and severe gastrointestinal bleeding to respiratory arrest and death.
Allele: One of two or more variants of a *gene* or other *DNA* sequence. Different alleles of a gene generally serve the same function (e.g., code for a *protein* that affects eye color) but may produce different *phenotypes* (e.g., blue eyes or brown eyes). Some alleles may be defective and produce a protein that has no function or an abnormal function.
Amino acids: A class of biological molecules, 20 of which serve as the building blocks of *proteins.*
Amygdala: A complex grouping of brain cells that, among other things, is thought to be involved in a person’s emotional reactions and in coordinating the body’s response to stress.
Bases: In genetics, the portion of a *nucleotide* molecule that contributes to the *genetic code*. *DNA* bases include adenine, thymine, guanine, and cytosine; in *RNA*, uracil replaces thymine.
Behavioral and cognitive deficits: Difficulties or disorders in social performance or learning, which include attention deficit disorder, behavioral undercontrol/conduct disorder, delinquency, lower IQ, poor school performance, and low self-esteem.
Binge drinking: The National Institute on Alcohol Abuse and Alcoholism defines binge drinking as the amount of alcohol leading to a blood alcohol content (BAC) of 0.08, which, for most adults, would be reached by consuming five drinks for men or four for women over a 2-hour period.
Cancellous bone: The type of tissue found in the marrow cavity of bone.
Candidate gene: A gene that has been implicated in causing or contributing to a particular *phenotype* (e.g., disease).
Catecholamine: One of a group of physiologically active substances with various roles in the functioning of the nervous system; also helps regulate heart functioning.
Catechol-*O*-methyltransferase (COMT): An *enzyme* that catalyzes the transfer of a methyl group to *catcholamines*, including the *neurotransmitters dopamine*, norepinephrine, and epinephrine.
Central nervous system (CNS): The part of the nervous system consisting of the brain and spinal cord.
Children of alcoholics: Biological children, foster children, adopted children, or stepchildren living in households with one or more adults classified as having an alcohol abuse or dependence diagnosis during the past year.
Chromosomes: Microscopic rod-shaped structures composed of double-stranded *DNA* and *proteins*; can be visualized during a certain phase of the cell cycle. Chromosomes are often regarded as representing the entire *genome* of an organism.
Cortical bone: Tissue that forms the shaft of the bone.
DNA (deoxyribonucleic acid): The molecule that carries the *genetic code* in all organisms except some viruses. DNA is composed of a linear sequence of *nucleotides*.
Dopamine: An excitatory *neurotransmitter* that plays a role in the reward system in the brain and possibly also in the reinforcing properties of alcohol use.
Endophenotype: A heritable trait or characteristic that is not a direct symptom of the condition under investigation but has been shown to be associated with the condition; for example, certain neurobiological characteristics have been noted in people with alcoholism and may be used as endophenotypes to identify people at risk for alcoholism.
Environmental-level interventions: Efforts to prevent alcohol use problems by focusing on changing the environment to reduce the availability of alcohol to youth and opportunities to drink, increase penalties for violation of minimum legal drinking age laws, and lower a community’s tolerance for alcohol use by youth.
Enzyme: A substance (usually a *protein*) that speeds up, or catalyzes, a specific biochemical reaction without being itself permanently altered or consumed.
Event-related potentials (ERPs): Brain waves elicited by a stimulus. One component of the ERP, measured approximately 300 milliseconds after exposure to the stimulus, is called the P300 signal. It is thought to represent cognitive processing of new information and is commonly reduced in people at risk for alcoholism.
Externalizing disorders: A constellation of behaviors including acting negatively on the external environment; and disruptive, hyperactive, and aggressive behavior. In the aggregate, these behaviors may also be referred to as conduct problems and antisocial or undercontrolled behavior.
G protein: Intracellular regulatory molecules whose actions are instigated by *neurotransmitters*. Several types of G proteins exist, including stimulatory G proteins (G_s_), which enhance the activities of *enzymes*, and inhibitory G proteins (G_i_), which inhibit the activities of other enzymes*.*
Gamma-aminobutyric acid (GABA): An inhibitory *neurotransmitter* whose actions are influenced by alcohol; may play a role in alcohol withdrawal.
Gene: A combination of *DNA* segments that together constitute a unit capable of expressing one or more functional gene products.
Gene expression: The processes through which the genetic information contained within a gene on the *DNA* is converted into a gene product (e.g., a *protein*).
Genetic code: The way in which information is carried by the *DNA* molecules determines the arrangement of *amino acids* in the *proteins* synthesized by the cells. Each of the 20 amino acids found in proteins is represented by 1 or more units of 3 consecutive *nucleotide bases* in the *mRNA* and in the *DNA* from which the *mRNA* is derived. All living organisms and viruses use the same genetic code.
Genetic marker: A segment of *DNA* with an identifiable physical location on a chromosome. The inheritance of a genetic marker can be followed.
Genome: The total genetic material of an organism or species.
Genomics: The comprehensive study of the interactions and functional dynamics of whole sets of *genes* and their products.
Genotype: The genetic makeup of an individual organism, which is determined by the specific *alleles* of each *gene* carried by the individual. Differences in alleles among individuals interact with environmental influences to account for the differences in *phenotype* observed among those individuals.
Glucocorticoid: Any of a group of steroid hormones, such as cortisol, that are produced by the adrenal gland and are involved in the metabolism of carbohydrates, *proteins*, and fats; plays a key role in the stress response.
Hypothalamic-pituitary-gonadal (HPG) axis: A system consisting of the hypothalamus, the pituitary, and either the ovaries or testes, which is involved in female or male reproduction, respectively, and awakens dramatically at puberty.
Indicated prevention: Prevention efforts that identify individuals who are exhibiting early signs of alcohol abuse and other associated problem behaviors and target them with special programs.
Individual-level interventions: Efforts to prevent alcohol use problems by focusing on the individual—that is, changing the person’s knowledge, attitudes, and skills, so that he or she is better able to resist influences that support drinking.
Insulinlike growth factor-1 (IGF-1): A *protein* produced by the liver in response to growth hormone (GH). IGF-1 carries out some of the effects of GH at the tissue level.
Internalizing disorders: A tendency in some children to express emotional distress within the child’s internal environment rather than his or her external world; behavior includes problems such as being withdrawn, anxious, inhibited, or depressed; also referred to as overcontrolled.
Knockout: The deletion or deactivation of a *gene* in a mouse or other laboratory animal to create a line of animals that are incapable of producing the *gene* product.
Mitochondria: Structures within cells that generate most of the cell’s energy through the production of *adenosine triphosphate (ATP)*.
Neuron: A nerve cell.
Neuropeptide: A small molecule that can regulate nerve cell function and is made up of *amino acids*.
Neurotransmitter: A chemical messenger (i.e., *dopamine, GABA*) that conveys a signal from one *neuron* to another.
Nucleotides: Biological molecules with a variety of physiologic and metabolic functions; nucleotides also serve as the building blocks of *DNA* and *RNA*.
Phenotype: The observable structural or functional characteristics of an individual organism that result from the interaction of its *genotype* with environmental factors.
Phosphorylation: A chemical reaction resulting in the addition of phosphate groups to other molecules (e.g., *proteins*). Phosphorylation reactions often are critical to regulation of *receptor* activity and the functions of other proteins.
Protein: The product of the genetic information encoded in a *gene*. Proteins are made up of *amino acids*; *enzymes* are one type of protein.
Protein kinases: *Enzymes* that add phosphate groups to other *proteins* in a chemical reaction called *phosphorylation*; this activates or inactivates the modified proteins.
Receptor: A *protein*, usually found on the surface of a *neuron* or other cell, that recognizes and binds to *neurotransmitters* or other chemical messengers.
Reinforcement: A process by which a response or behavior (e.g., alcohol consumption) is strengthened by the anticipation of a reward (e.g., a feeling of euphoria).
Ribonucleic acid (RNA): A class of molecules composed of *nucleotides*, similar to those that form *DNA*. The major types of RNA are mRNA, tRNA, and rRNA, which play important roles in *gene expression*.
Selected prevention: Prevention strategies that target subsets of the total population which are deemed to be at risk for alcohol problems by virtue of their membership in a particular population segment—for example, *children of adult alcoholics*, dropouts, or students who are failing academically.
Self-regulation: The ability to monitor and modulate internal states, including both the ability to modulate affect and level of arousal and the neurocognitive executive capacities to engage in goal-directed behavior. These executive cognitive capacities include the regulation of attention, planning, organization, concept formation, abstract reasoning, cognitive flexibility, self-monitoring, motor programming, and motor control.
Serotonin: A *neurotransmitter* that subtly modifies *neuron* function, exerting its effects by interacting with *receptors* on the neuron’s surface.
Synapse: A microscopic gap separating adjacent *neurons,* where *neurotransmitters* and *receptors* cluster.
Transgenic animals: An animal into whose *genome foreign DNA* (i.e., a *candidate gene*) has been introduced to study the function of *DNA*.
Universal prevention: Prevention strategies that address the entire population (national, local community, school, neighborhood) with messages and programs aimed at preventing or delaying the abuse of alcohol.

